# X-ray structures of fructosyl peptide oxidases revealing residues responsible for gating oxygen access in the oxidative half reaction

**DOI:** 10.1038/s41598-017-02657-5

**Published:** 2017-06-05

**Authors:** Tomohisa Shimasaki, Hiromi Yoshida, Shigehiro Kamitori, Koji Sode

**Affiliations:** 1 Department of Biotechnology and Life Science, Graduate School of Engineering, Tokyo University of Agriculture and Technology, 2-24-16, Nakamachi, Koganei, Tokyo 184-8588 Japan; 2 Life Science Research Center and Faculty of Medicine, Kagawa University, Ikenobe, Miki-cho, Kita-gun, Kagawa Japan

## Abstract

Current enzymatic systems for quantifying glycated hemoglobin are based on the FAD-containing enzyme fructosyl peptide oxidase (FPOX). FPOX has substrate specificity for fructosyl-^α^ *N*-valyl-histidine derived from proteolytic digestion of the N-terminus of the HbA1c β-chain. This study reports the X-ray structures of the wild-type and Asn56Ala (N56A) mutant of *Phaeosphaeria nodorum* fructosyl peptide oxidase (PnFPOX) to elucidate the residues responsible for the oxidative half-reaction. N56A showed decreased oxidase activity compared to the wild -type, while its dye-mediated dehydrogenase activity was higher than that of wild type. In wild-type PnFPOX, Asn56 forms a hydrogen bond with Lys274, thereby preventing it from forming a salt bridge with Asp54. By contrast, Lys274 of PnFPOX N56A moves toward Asp54, and they approach each other to form a salt bridge at a distance of 2.92–3.35 Å. Site-directed mutagenesis studies and protein channel analysis suggest that Asp54 assists in accepting oxygen properly at the position of the bound water molecule in the main oxygen channel. These results reveal that Asn56 in PnFPOX is essential for maintaining an effective oxygen accession path, and support the role of Asp54 as a gate keeper that cooperates with Lys274 to enable oxygen to reach the active site properly.

## Introduction

**Figure 1:**
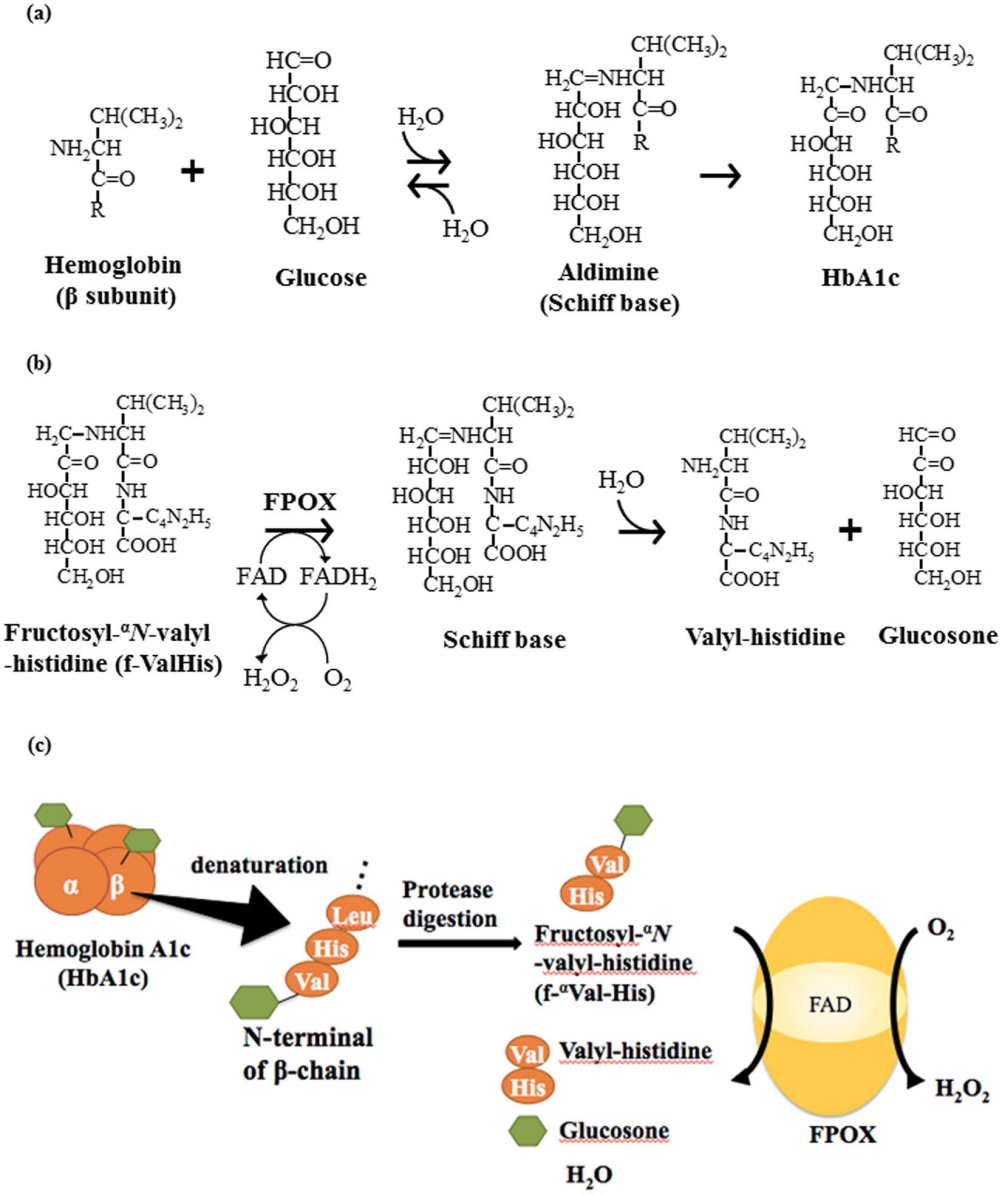
Reaction scheme of non-enzymatic HbA1c-formation reaction, the catalytic reaction of FPOX, and representative principle of HbA1c measurement using FPOX. (**a**) Non-enzymatic reaction between glucose and the N-terminal valine of the hemoglobin β subunit, resulting in HbA1c. (**b**) Oxidation of fructosyl-^α^ *N*-valyl-histidine (f-^α^Val-His) is catalyzed by FPOX. The C-N bond linking the C1 of the fructosyl moiety and nitrogen of the amino group is oxidized. The resulting Schiff base is rapidly hydrolyzed and glucosone and valyl-histidine are produced. (**c**) HbA1c in the blood is denatured and then digested by protease to release f-^α^Val-His. f-^α^Val-His is oxidized by FPOX.

Maintenance of optimal blood glucose levels is an essential treatment module for patients with diabetes mellitus to avoid long-term complications. Non-enzymatic reactions between glucose and various circulating blood proteins, such as hemoglobin and albumin, result in the formation of glycated proteins. Glycated hemoglobin, also known as hemoglobin A1c (HbA1c) and glycated albumin (GA), are important indicators of glycemic control in diabetes mellitus. They are also used as markers for assessing the effectiveness of diabetes treatment. HbA1c originates from the glycation of the N-terminal valine of the hemoglobin β subunit and reflects the mean blood glucose level over the past 2–3 months^1, 2^ (Fig. 1(a)). GA originates from the glycation of the ε-amino group of internal lysine residues of circulating albumin in plasma and reflects the average blood glucose level over the past 2–3 weeks. The conventional methods for measuring HbA1c and GA are based on high performance liquid chromatography (HPLC) or immunoassay techniques. Although the HPLC assay method is characterized by high precision and high accuracy, the methods involve expensive equipment, requiring specially trained staff and relatively long operating times. By contrast, an immunoassay method is included in clinical chemistry analyzers, and is expected to be easily applied in the measurement of large volumes of samples at the same time. However, the immunoassay method currently in use, measures the change in the absorption caused by the aggregations produced by antibody-antigen reactions, and measurement precision and accuracy are lower than those of the HPLC assay method. To overcome the drawbacks of HPLC and immunoassay methods for glycated proteins, convenient and reproducible assay systems based on enzyme activity have become attractive alternatives to conventional detection methods^3–5^.

These enzyme assay systems are based on the enzyme fructosyl amino acid oxidase (FAOX) or fructosyl peptide oxidase (FPOX). Fructosyl amine oxidases (FAOXs) are FAD-containing enzymes that catalyze the oxidative deglycosylation of fructosyl amino acids such as fructosyl valine and/or ε-fructosyl lysine, which are the degradation products of HbA1c and GA, respectively. As members of the FAOX family, fructosyl peptide oxidases (FPOXs) are also known as candidates for diagnostic enzymes^6^. A few promising FPOXs derived from *Eupenicillium terrenum* (EtFPOX) and *Coniochaeta* sp. (FPOX-C) have been reported^7^. Figure 1(b) shows the scheme of the FPOX catalyzed reaction. FPOX has substrate specificity for fructosyl-^α^ *N*-valyl-histidine (f-^α^Val-His) derived from the glycated N-terminal residues of the HbA1c β-chain. Figure 1(c) shows the representative procedure for the enzyme assay for HbA1c using FPOX. HbA1c is digested by protease to release f-^α^Val-His. FPOX, which catalyzes the oxidation of f-^α^Val-His, and hydrogen peroxide is produced. Subsequently, the liberated hydrogen peroxide is measured using a conventional peroxidase -based color -developing method. This enzymatic assay is optimized to formulate reagents that are ready to use in conventional autoanalyzers. FAOX/FPOX-based measurement systems are expected to become a major component of glycated protein sensing since they are rapid, reproducible, and suitable for automated analyzers.

HbA1c is the standard accepted indicator of glycemic control because sufficient evidence has been established to support the relationship of HbA1c and diabetes-associated complications. There has been continued interest in searching for novel FAODs/FPOXs. We reported on the identification of a novel FPOX from *Phaeosphaeria nodorum* (PnFPOX)^8^. PnFPOX showed a remarkably low *K*_*m*_ value for f-^α^Val-His (0.185 mM), distinguishing it from previously reported FPOXs^7^. Furthermore, PnFPOX showed higher thermal stability than other FPOXs. These unique features of PnFPOX are very advantageous for its application to the enzymatic measurement of HbA1c.

At present, the crystal structures of only a few FAOX family members are known. The first FAOX to be determined was amadoriase II, i.e., FAOX derived from *Aspergillus fumigatus* ^9^. The structures of FPOX derived from *Eupenicillium terrenum* (EtFPOX)^10^ and amadoriase I (FAOX derived from *Aspergillus fumigatus*)^11^ have since been reported. These enzymes have different substrate specificities, and the features giving rise to these differences are now understood based on their structures. Amadoriase II has a deep hydrophobic channel at the catalytic site, formed by the ordering of flexible loops. This enzyme is active not on fructosyl peptides but rather on fructosyl amino acids. By contrast, the cavity of the catalytic site in EtFPOX has a wide opening that accommodates peptide substrates, such that it is active on α-fructosyl peptides. Amadoriase I shows a tunnel conformation leading to the catalytic site through a narrow passage; four gating loops and helices are present at the entrance, restricting the access of fructosyl amino acids with charged side chains. These extensive studies on substrate recognition during the reductive half reaction will result in further advancements in the development of enzymes with superior and favorable substrate specificities that can be used for clinical analyses. Putative catalytic mechanisms for FAOX were reported previously^12^. The kinetic data were consistent with a three-step reductive half-reaction in which the oxidized enzyme binds the substrate, flavin reduction occurs, and product is released. In the absence of a full steady-state analysis of FAOX, it is not known whether a ternary complex forms during turnover. However, a similar mechanism to MSOX^13^ was proposed for FAOX in which catalysis proceeds via a “modified” ping pong mechanism; oxygen reacts with reduced status prior to the dissociation of the product. Because most of residues postulated to be involved in the catalytic mechanism are conserved between FAOX and FPOX, the same catalytic mechanisms for FAOX will proceed in FPOX. However, only limited information is available on the structure-function relationship in the oxidative half reaction of FAOX/FPOX. Such information is needed to understand the preferred electron acceptor; and this information is required for designing and/or choosing adequate electron acceptors for the detection of an enzyme reaction.

We have been engaged in engineering the conversion of FAD-dependent oxidases to dehydrogenases to create engineered oxidases that are not affected by the presence of oxygen when they are utilized as enzymes in electron mediator type enzyme electrodes. These studies were performed using amino acid residues substitutions, at positions predicted to be responsible for oxygen binding or recognition. Cholesterol oxidase^14^ and glucose oxidase^15, 16^, members of the glucose-methanol-choline oxidoreductase family, have been engineered. The engineered oxidases exhibit drastically lower oxidase activity but equivalent or even increased dye-mediated dehydrogenase activities. We also reported amino acid substitutions in FAOX and FPOX to yield the mutants FAOX^17^ and FPOX^18^, which were essentially converted into dehydrogenases by these alterations. For this engineering study, we used PnFPOX^8^. The mutant Asn56Ala (N56A) showed lower oxidase activity when oxygen was used as the electron acceptor, while its dye-mediated dehydrogenase activity was higher than that of wild-type^18^. Therefore, elucidation of the structures of the wild-type and Asn56Ala mutant PnFPOX will provide significant information needed to understand the oxidative half-reaction of this enzyme group.

In this study, we report the X-ray structures of the recombinant wild-type and N56A PnFPOX. Comparison of the structures at the *si*-face of FAD in PnFPOXs showed an intriguing difference between the wild type and N56A. In the structure of wild-type PnFPOX, Asn56 forms a hydrogen bond with Lys274, preventing the formation of salt bridges with Asp54, which leads to the creation of a space. In the N56A mutant, Lys274 moves toward Asp54 to form salt bridges and occupies the space, which leads to a decrease in the oxygen activity as shown in our previous mutagenesis study. Site directed mutagenesis studies suggested an intriguing role for Asp54 in the oxidative half reaction. Our structural comparison and analysis of the protein channel revealed that Asn56 in PnFPOX plays a crucial role in maintaining a plausible main oxygen channel with Asp54 and Lys274 for oxidase activity.

## Results

### Overall structure of PnFPOX

**Figure 2:**
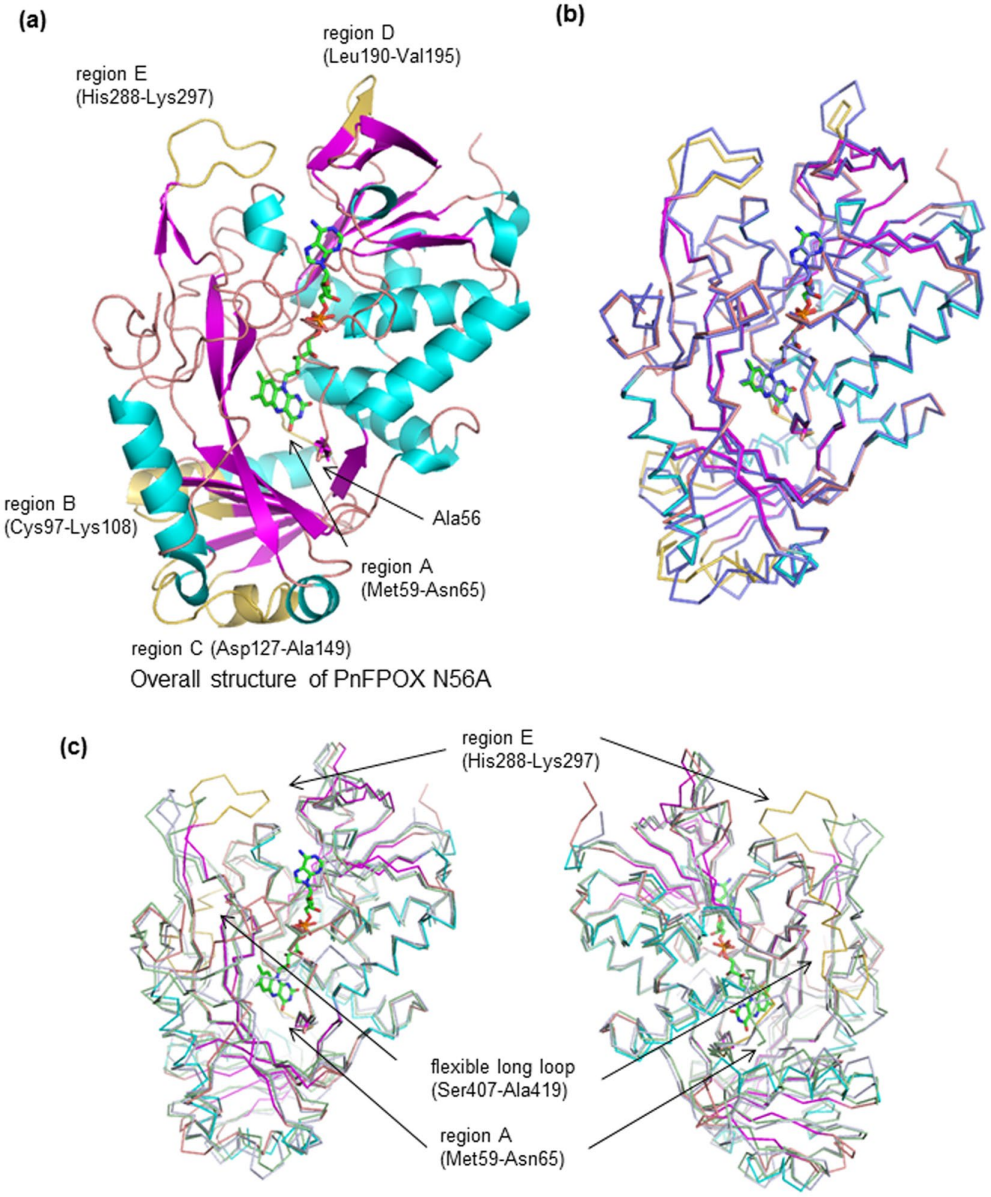
Structural comparison between wild-type PnFPOX, EtFPOX, and FAOXs. (**a**) Overall structure of wild-type PnFPOX. (**b**) Overall structure of EtFPOX (PDB: 4RSL, light blue) superimposed onto that of wild-type PnFPOX. Slight differences between the two models are colored in yellow in wild-type PnFPOX. The FAD of PnFPOX is shown as a stick model. (**c**) Overall structures of amadoriase I (PDB: 4XYZ, light green) and amadoriase II (PDB: 3DJE, light gray) superimposed onto wild-type PnFPOX. Remarkably different regions between the three models are colored in yellow in wild-type PnFPOX. FAD is shown as a stick model. The figure on the right is rotated 180 degrees along the y axis.

Since the overall structure of wild-type PnFPOX is equivalent to that of PnFPOX N56A, only the overall structure of wild-type PnFPOX is shown in Fig. 2(a). Compared to the structure of FPOX obtained from *Eupenicillium terrenum* (EtFPOX, PDB code: 4RSL), wild-type PnFPOX is structurally similar with 73% identity, 1.0 Å rmsd, and a 63.9 Z-score, as shown by a Dali search (Fig. 2(b)). Other structurally similar enzymes are amadoriase II without ligand (3DJD, 36% id, 1.8 Å rmsd, 49.8), amadoriase II with bound inhibitor (FSA) (3DJE, 35% id, 1.9 Å rmsd, 49.5), amadoriase I with bound substrate (fructosyl ^ε^lysine) (4XWZ, 33% id, 1.9 Å rmsd, 49.4), amadoriase I without ligand (4WCT 33% id, 1.9 Å rmsd, 49.2), and monomeric sarcosine oxidase (3M13, 25% id, 2.4 Å rmsd, 40.0).

The regions with slight differences between wild-type PnFPOX and EtFPOX are shown in Fig. 2(a). Five such regions were identified as follows: A (Met59-Asn65), B (Cys97-Lys108), C (Asp127-Ala149), D (Leu190-Val195), and E (His288-Lys297). Compared to amadoriase I and II, remarkable differences were observed in region A, in which amadoriase I and II have a longer loop 1 (Met59-Ser72 and Ile55-Ile67, respectively). In region E, amadoriase I and II have a shorter flexible loop 2 (Pro294-Glu302 and Glnl289-Met295, respectively), and a long flexible loop (Ser407-Ala419) of PnFPOX corresponding to Thr407-Met426 and Ile399-Met419 in amadoriase I and II, respectively (Fig. 2(c)).

### FAD binding site

A covalent bond is formed between FAD and Cys343 of PnFPOX as observed in amadoriase I (Cys342), amadoriase II (Cys335), and EtFPOX (Cys347). The residues, Thr18, Asp41, Ser47 and Ser50 (Ala49 in amadoriase I), Ala51 (main chain), Lys57 and Ile58 (main chain), and Lys376 (Met375 in amadoriase I), which form hydrogen bonds with FAD are almost conserved in FPOX and FAOX. In addition, Lys57 and Lys274 which approach the isoalloxazine ring of FAD at the *si*-face, are also conserved in FPOX and FAOX (Supplementary Figure S1). Since the substrates of the FAOX family enzymes bind at the *re*-face of the isoalloxazine ring of FAD, the FAD binding mode and environment at the *si-*face of the isoalloxazine ring do not affect substrate specificity and are conserved among the FAOX family members.

### Substrate binding site

**Figure 3:**
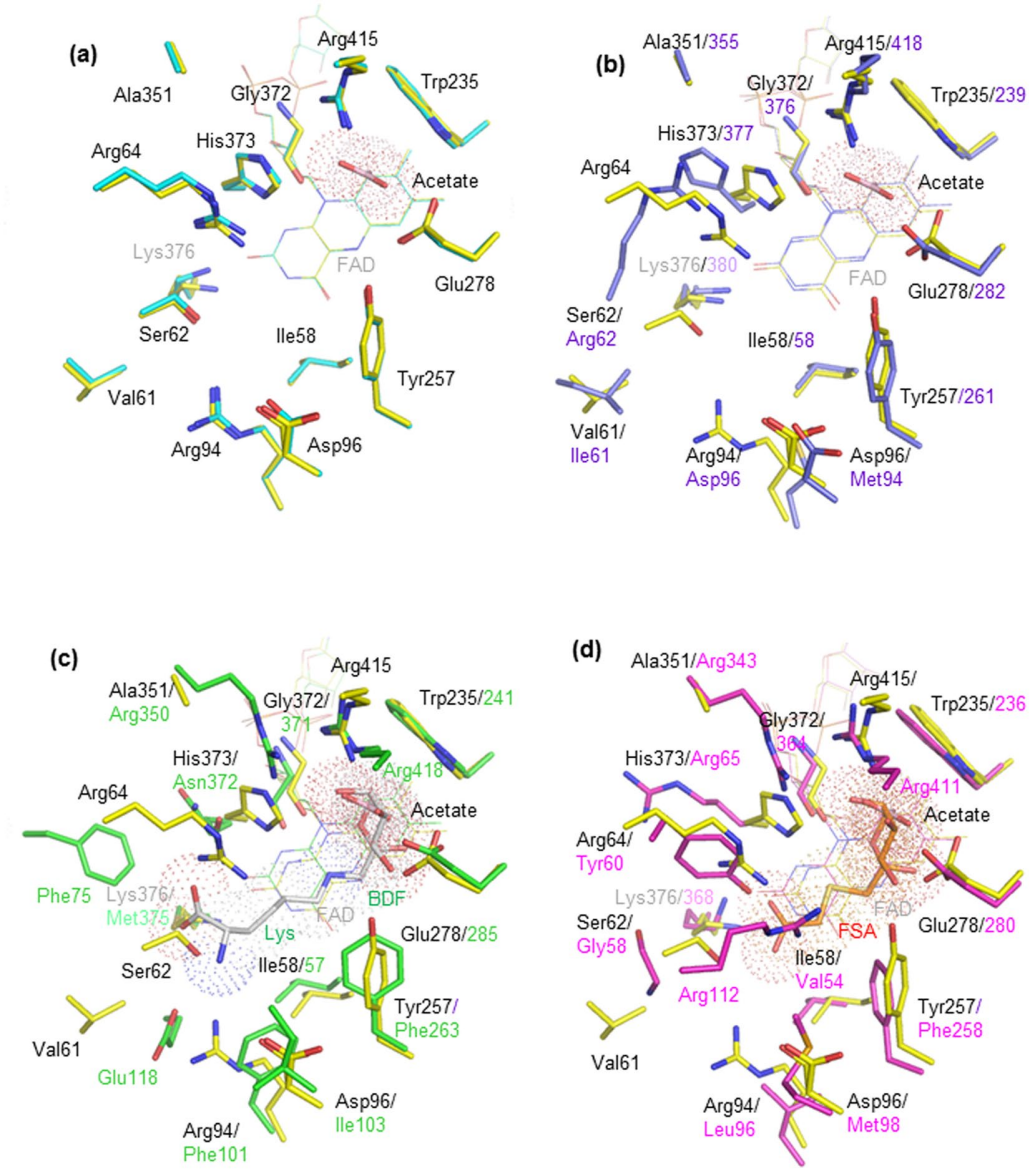
Substrate binding-site comparison of wild-type PnFPOX, N56A, EtFPOX, amadoriase I with bound BDF-Lys, and amadoriase II with bound FSA. The structures of (**a**) wild-type PnFPOX (yellow stick) with bound acetate (light pink stick with dots) and N56A (cyan stick), (**b**) wild-type PnFPOX with bound acetate and EtFPOX (purple stick) (PDB: 4RSL), (**c**) wild-type PnFPOX with bound acetate and amadoriase I (green stick) with bound fructosyllysine (white stick with dots) (PDB: 4XWZ), and (**d**) wild-type PnFPOX with bound acetate and amadoriase II (magenta stick) with bound FSA (orange stick with dots) (PDB: 3DJE). BDF: beta-D-fructopyranose. Lys: Lysine. FSA: 1-s-(carboxymethyl)-1-thio-beta-D-fructopyranose.

Figure 3 compares the substrate binding sites among the enzymes. Acetate molecules from the reservoir solution are bound to PnFPOX. A bound acetate to wild-type PnFPOX is located at the *re*-face of the isoalloxazine ring of FAD (Fig. 3(a)). No acetate molecule was found in the active site of PnFPOX. Comparison of active site structures between wild-type PnFPOX with bound acetate and PnFPOX N56A revealed no significant differences (Fig. 3(a)). The active site of EtFPOX is superimposed onto that of wild-type PnFPOX/acetate as shown in Fig. 3(b). Near the bound acetate, the orientation of the side chains of Glu278 and His373 differs from those of the conserved residues in EtFPOX. The residues Val61, Ser62, Arg64 (corresponding to region A in Fig. 2(a)), Arg94 and Asp96 followed by region B in Fig. 2(a), are not conserved in EtFPOX (Fig. 3(b)). The positions of the side chains of Ser62, Arg64, Arg94 and His373 create a smaller cavity for accepting and recognizing substrates in PnFPOX compared to EtFPOX. Figure 3(c) and (d) shows the superimposed active site structures of amadoriase I with bound BDF-Lys (fructosyl-^ε^ *N*-lysine, f-^ε^Lys) and amadoriase II with bound FSA (fructosyl thioacetate; inhibitor) onto the active site of wild-type PnFPOX/acetate, respectively. The bound acetate of wild-type PnFPOX corresponds to the position of bound beta-D-fructopyranose (BDF) in amadoriase I (Fig. 3(c)) and the position of FSA in amadoriase II (Fig. 3(d)). The positions of the O1, O2, and CH3 of the bound acetate correspond to the O5, C4, and C6, respectively, of the bound BDF and the fructosyl moiety of the bound FSA (Supplementary Figure S2). This suggests that the position of the bound acetate is the binding site of a region of the fructosyl moiety of the substrate (fructosyl amino acids). Indeed, Trp235, Glu278, and Gly372 of PnFPOX are conserved in amadoriases I and II, whose corresponding residues are involved in recognizing the fructosyl moiety of the substrate. In addition, the NH2 and NE of Arg418 in the long flexible loop of amadoriase I (NH2 and NE of Arg411 in amadoriase II) also interact with the fructosyl moiety, whereas the corresponding positions for fructose interaction are covered by the NH1 and NH2 of Arg415 in PnFPOX. Thus, PnFPOX would recognize the fructosyl moiety of the substrate through these conserved residues.

Most residues in the active site are not conserved between PnFPOX and amadoriase I, except for the residues recognizing the fructosyl moiety (Fig. 3(c)).

The positions of the side chains of Val61, Ser62, Arg64, Arg94, Asp96, Ala351, and His373 in PnFPOX differ from those in the corresponding area of amadoriase I. Ser62 of PnFPOX causes steric hindrance at the position of the bound Lys portion of f-^ε^Lys in amadoriase I/BDF-Lys, suggesting that f-^ε^Lys is not well accepted as a substrate by PnFPOX (Fig. 3(C)). Arg64 is also present at a closer proximity to the Lysine moiety of f-^ε^Lys. Similar significant differences in the active sites between PnFPOX and amadoriase II are shown in Fig. 3(d). Ile58, Val61, Ser62, Arg64, Arg94, Asp96, Tyr257, Ala351, and His373 in PnFPOX are not conserved in amadoriase II, leading to differently-sized cavities for accepting the amino acyl moiety of the substrate. These residues of PnFPOX can also accept FSA (inhibitor, the analog of f-^α^Val) in the cavity without any steric hindrance and would have a wide cavity for the substrate, since there is no residue corresponding to the position of Arg112 in amadoriase II. These observations explain why PnFPOX preferentially recognizes ^α^-glycated amino acids such as fructosyl-^α^ *N*-valine (f-^α^Val) or fructosyl-^α^ *N*-valyl-histidine (f-^α^Val-His) as substrates, but not ε-glycated amino acids such as fructosyl-^ε^ *N*-lysine (f-^ε^Lys). The differences in cavity size and shape formed by different amino acid residues account for the differing substrate specificities among the FAOX family members.

### Role of Asn56

**Figure 4:**
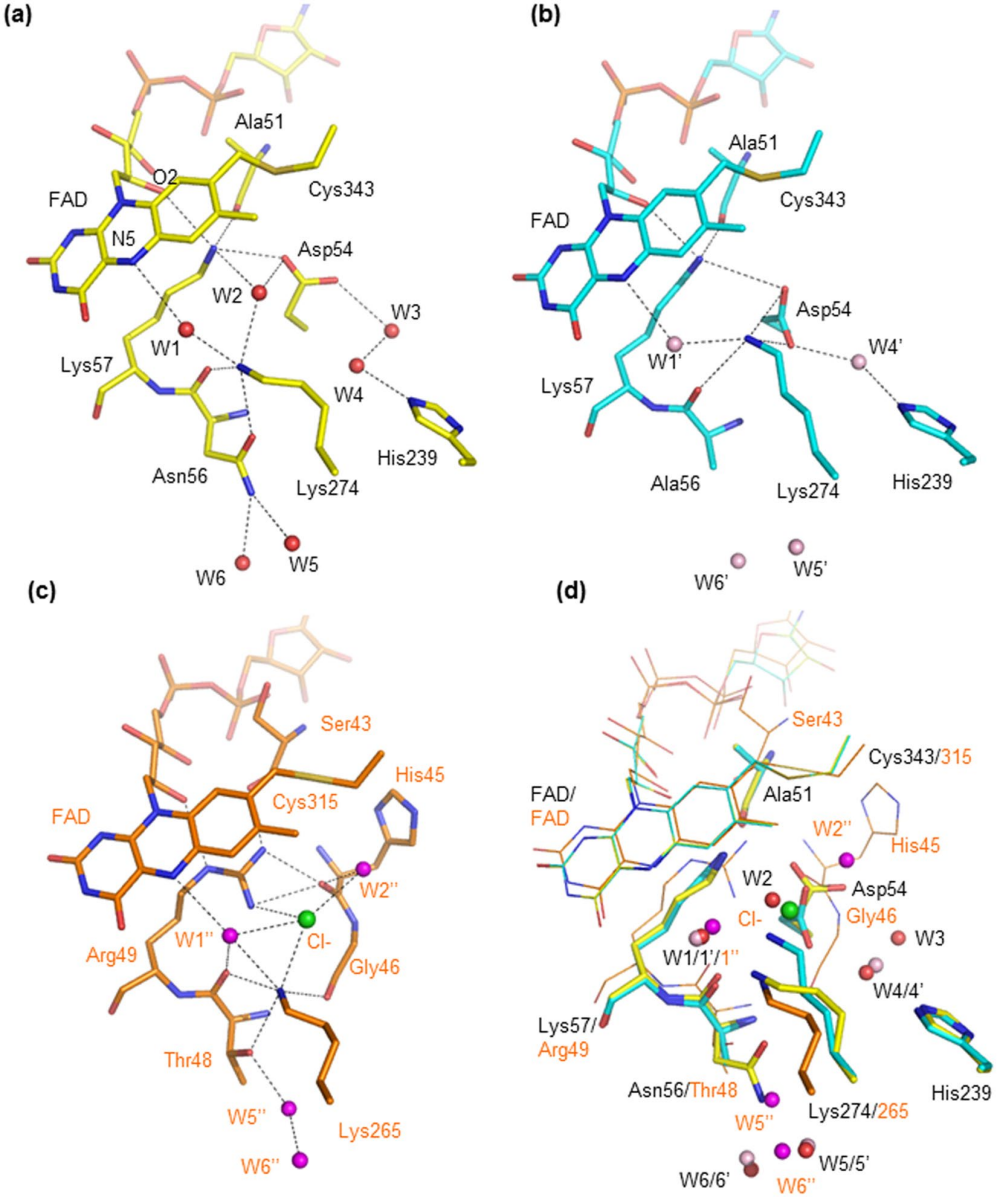
Comparison of structures around FAD at the *si*-face of wild-type PnFPOX, N56A, and the oxygen binding site of monomeric sarcosine oxidase (MSOX) with bound chloride. (**a**) Structure of wild-type PnFPOX. Red spheres indicate water molecules. (**b**) Structure of PnFPOX N56A. Light pink spheres indicate water molecules. (**c**) Structure of MSOX with bound chloride (PDB: 3QSM). Light green sphere indicates chloride. Magenta spheres indicate water molecules. (**d**) The structures of (**b**) and (**c**) were superimposed onto that of (**a**). FAD and covalently bonded Cys, and the structure of MSOX are shown as a line model. Selected hydrogen bonds and salt bridge interactions (<3.53 Å) are indicated by dotted lines. Dotted lines indicate the distance between selected atoms.

In wild-type PnFPOX, Asn56 forms a hydrogen bond with Lys274; Lys274 forms water (W1)-mediated hydrogen bonds with N5 of FAD and hydrogen bonds with Asp54 and Lys57 and O2 of FAD via a water molecule (W2) (Fig. 4(a)). Although the electron density maps of Asp54 indicate the possibility of an alternative conformation (Supplementary Figure S3(a)), the distance between W2 and OD2′ of Asp54 in the alternative conformation is too short (1.1 Å). The position of W2 and conformation of Asp54 were determined from the sa-omit maps at 3.5–4.0 σ (Supplementary Figure S3(b,c)). In PnFPOX N56A, Lys274, and Asp54 approach each other, and the water molecule is displaced from the W2 position such that Lys274 forms salt bridges with Asp54 at 2.92–3.35 Å (Supplementary Figure S4(b)). The direct distance between Lys274 and Asp54 is 5.48–6.03 Å in wild-type PnFPOX (Supplementary Figure S4(a)). The relocated Asp54 also forms water (W3′ and W4′)-mediated hydrogen bond with His239 and is stabilized in this position. Considering that PnFPOX N56A has lower oxidase activity and a concomitant increase in dehydrogenase activity^18^, the role of Asn56 is likely to keep Lys274 free, and to prevent the formation of salt bridges with Asp54, thereby creating a space for accepting a significant water molecule at the W2 position.

### Site-directed mutagenesis studies on Asp54

From the X-ray structures, we found an unexpected movement of Asp54 in the mutant N56A. To investigate the role of Asp54, we conducted site-directed mutagenesis at Asp54. Eight PnFPOX Asp54 variants, D54E/N/A/H/V/S/F/Y, were constructed. These mutants showed significant decreases in both oxidase and dehydrogenase activities (Supplementary Table S1). These drastic decreases in the enzymatic activities of mutant enzymes did not correlate to the expression level of mutant enzymes, except for D54Y and D54F. The expression levels of D54E, D54N, D54A, D54H, D54V and D54S as soluble proteins were 50%, 60%, 110%, 80%, 60% and 110% of wild type, respectively (Supplementary Figure S5; these values are calculated based on the image analyses of band intensity using ImageJ software (NIH, Bethesda, MD, USA)). However, the oxidase activities of D54E, D54N, D54A, D54H, D54V and D54S were 1.2%, 1.8%, 5.1%, 3.0%, 0.11% and 4.1% of wild type, respectively (Supplementary Table S1). These results indicated that the drastic decrease in the enzyme activity of these mutants were not due to the low level of expression as the soluble proteins, but due to the change in the enzymatic activity. More remarkably, the effect was much greater with respect to oxidase activity than dehydrogenase activity. These results suggest that Asp54 plays a significant role in the oxidative half-reaction, especially in the reaction involving oxygen as the electron acceptor.

In our previous saturation mutagenesis study on Asn56 in non-his-tagged PnFPOX, Asn56 was found to be involved in oxidase activity and the putative proton relay system^18^. Therefore, we combined the Asn56Ala mutation with Asp54 mutations. Among constructed Asp54 variants, Asp54Val showed the highest level of dehydrogenase activity. However, the double mutant, D54V/N56A was not properly expressed in *E. coli* as a soluble and active enzyme, but instead formed inclusion bodies. The reason for this observation has yet to be investigated; however, these mutations are likely incompatible with the proper folding of the molecule. By contrast, the other mutants were expressed and showed detectable oxidase and dehydrogenase activity. Interestingly, PnFPOX D54A/N56A, D54N/N56A, D54E/N56A, D54H/N56A and D54F/N56A showed higher levels of dehydrogenase activity and lower levels of oxidase activity than single mutants. The oxidase activity of D54E/N56N was about 4 times or higher than the oxidase level of D54N/N56A and D54A/N56A. Among these mutants PnFPOX D54A/N56A and D54N/N56A showed the highest Dh/Ox values. Therefore, these two double mutants were characterized using purified enzymes (Table 1). D54A/N56A and D54N/N56A had 11% and 4.3% of the *V*_max_/*K*_m_ for oxidase activity relative to N56A, and 7.2% and 2.9% relative to wild type. However, the *V*_max_/*K*_m_ for dye-mediated dehydrogenase activity was 21% and 20% for D54A/N56A and D54N/N56A compared to N56A, and 41% and 39% compared to wild -type. It is remarkable that the D54A and D54N amino acid substitutions resulted in drastic decreases in both oxidase and dehydrogenase activity. However, in combination with the N56A mutation, the effect on the decrease in dehydrogenase activity was limited. Consequently, the dehydrogenase/oxidase ratios based on *V*_max_/*K*_m_ values for D54A/N56A and D54N/N56A were 910% and 386%, respectively; these values are much higher than those of N56A (192%) and wild type (67%). Together with the results of single mutations of Asp54, these results suggest that Asp54 is also involved in the oxidative half-reaction, mainly in oxidase activity.

**Table 1:** Kinetic parameters of wild-type PnFPOX, N56A, D54A/N56A and D54N/N56A.

	Oxidase activity (Ox)			dye-mediated dehydrogenase activity (Dh)			Dh/Ox
	*K*_m_ [mM]	*V*_max_ [U·mg^−1^]	*V*_max_/*K*_m_ [A] [U·mg^−1^·mM^−1^]	*K*_m_ [mM]	*V*_max_ [U·mg^−1^]	*V*_max_/*K*_m_ [B] [U·mg^−1^·mM^−1^]	[B]/[A]
Wild-type	0.359 ± 0.0062	29.9 ± 0.56	83.3	0.443 ± 0.042	24.5 ± 0.71	55.2	0.663
N56A	0.109 ± 0.0062	5.47 ± 0.068	50.2	0.67 ± 0.032	74.6 ± 1.3	111	2.20
D54N/N56A	0.046 ± 0.0037	0.109 ± 0.0024	2.38	1.33 ± 0.027	28.7 ± 0.25	21.7	9.12
D54A/N56A	0.014 ± 0.0012	0.078 ± 0.0017	5.73	0.92 ± 0.024	20.9 ± 0.21	22.7	3.96

## Discussion

This study determined the X-ray structures of both wild-type and a mutant PnFPOXs to understand the structure-function relationship of the oxidative half reaction of FAOX and FPOX. A comparison of the PnFPOX wild-type and mutant N56A X-ray structures clearly revealed the crucial role of Asn56 in oxidase activity, and we propose that it indirectly contributes by accepting an important water molecule at the W2 position.

In wild-type PnFPOX, Asn56 forms a hydrogen bond with Lys274 (2.93 Å), which interacts with N5 of the isoalloxazine ring of FAD by a water-mediated hydrogen bond. The water molecule (W1) is located at the *si*-face of the isoalloxazine ring 2.51 Å from Lys274 and 3.22 Å from N5 of the isoalloxazine ring. In addition, Lys274 interacts with Asp54 and Lys57 via a water molecule (W2) (Supplementary Figure S4(a)). Interestingly, in PnFPOX N56A, Lys274 cannot form a hydrogen bond with Ala56 but instead moves toward Asp54, forming salt bridges 2.92–3.35 Å in length (Supplementary Figure S4(b)). This movement results in the elimination of the water molecule from position W2, although Lys274 can interact with N5 of the isoalloxazine ring via a water molecule (W1, 2.81 Å from Lys274 and 3.11 Å from N5 of the isoalloxazine ring). The mutagenesis studies and crystal structure analyses of monomeric sarcosine oxidase (MSOX) suggest the importance of residues Arg49, Lys265, and two water molecules (corresponding to Lys57, Lys274, W1, and W2 of PnFPOX, respectively) in the active site of MSOX^13, 19–22^. The position of the oxygen-binding site has been discussed with respect to the structure of MSOX in a complex with chloride, which acts as an oxygen surrogate^22^. The bound chloride is in a small pocket instead of one of the water molecules that occupies the structure of MSOX alone. Figure 4(c) shows the active-site structure of MSOX with bound chloride (PDB: 3QSM). The position of the bound chloride was reported as the oxygen-binding site of MSOX^22^. The active-site structure of MSOX/chloride was superimposed on those of wild-type PnFPOX and N56A, as shown in Fig. 4(d). The positions of FAD, W1 interacting with N5 of the isoalloxazine ring, and Lys274 in wild-type PnFPOX are well conserved in MSOX. Interestingly, the related positions of Lys57 and W2 in wild-type PnFPOX correspond to those of Arg49 and the bound chloride in MSOX, suggesting that the W2 position might be a putative oxygen-binding site in PnFPOX (Fig. 4(d)). However, with the movement of Asp54 and Lys274 in PnFPOX N56A, the corresponding space no longer provides enough room to bind with a water molecule (W2). Since PnFPOX N56A exhibited decreased oxidase activity with a concomitant increase in dehydrogenase activity in our previous mutagenesis study^18^, this structural comparison with MSOX supports the idea of a putative oxygen-binding site of PnFPOX. The role of Asn56 appeared to maintain the freedom of Lys274, and did not form salt bridges with Asp54, which maintains a space for accepting oxygen at the W2 position. The observed increase in dye-mediated dehydrogenase activity in PnFPOX N56A can be explained by the decrease in competition between the dye mediator and oxygen. To investigate the oxygen binding site of PnFPOX, we attempted to soak NaCl into the crystals of wild-type and N56A PnFPOX, since the bound chloride side is considered an oxygen binding site in MSOX as described above. However, no bound chloride was confirmed in NaCl soaked crystals of wild-type PnFPOX at this resolution (3.08 Å) (Supplementary Table S2). The crystal of PnFPOX N56A with NaCl diffracted well and the structure was determined at 1.8 Å. Although bound chloride ions were found in the structure of PnFPOX N56A with NaCl, no chloride was bound at the W2 position (Supplementary Table S2). Oxygen was almost unable to bind at the position of W2. Next, we analyzed the protein channels of the structures of wild-type PnFPOX, N56A, and structures with NaCl using the program CAVER 3.0 (http://www.caver.cz/)^23^. The analysis of the protein channels of the structures showed that the W1, W2, and W4 positions in wild-type PnFPOX (W1′ and W4′ in PnFPOX N56A) are in the main oxygen-accessible channel (Supplementary Figures S6, S7, and S8). In PnFPOX N56A, the movement of Asp54 and Lys274 blocks a short and plausible main channel observed in wild-type PnFPOX (blue dotted path with arrow in Supplementary Figure S6). Although the oxygen could be accessible through another alternative long path instead of the closed path in PnFPOX N56A, the elimination of the accessibility toward the short pathway might result the drastic decrease in oxidase activity compared with dye-mediated dehydrogenase activity. Therefore, we concluded that W2 could not be a putative oxygen binding site, but that the W2 position is in the plausible main oxygen accessible channel. The role of Asp54 is as a gate keeper that cooperates with Lys274 to enable oxygen to reach the active site properly.

In a previous study, we reported that the three residues, Asn56, Lys57, and Lys274 in PnFPOX were conserved in a putative proton relay system (PRS) between FPOX, amadoriase I and II, and monomeric sarcosine oxidase^17^. In MSOX, a hydrogen-bonded network extending from N5 of FAD to the protein surface is focused on as a part of the PRS. The PRS involves Thr48, Lys265, and four water molecules^24^. A structural comparison of wild-type PnFPOX and N56A suggests that Asp54 may also have a crucial role in the oxidative half reaction. Indeed, site-directed mutagenesis studies of Asp54 resulted in a mutant-enzyme with drastic decreases in both oxidase and dehydrogenase activities, with higher dehydrogenase/oxidase ratios compared to the wild-type. Because Lys274 forms hydrogen bonds with Lys57 via W2 and forms hydrogen bonds with FAD via W1, the replacement of Asp54 with Asn or Ala could influence the suitable positions of Lys57 and Lys274 or disturb the proton relay system. Therefore, Asp54 mutagenesis might lead to decreases in both oxidase activity and dye-mediated dehydrogenase activity. However, the combination of the Asp54 mutation with the Asn56Ala mutation resulted in a drastic increase in the dehydrogenase/oxidase ratio compared to that of N56A and wild-type. This change was caused by a drastic decrease in oxidase activity, resulting in an apparent increase in the dye-mediated dehydrogenase activity. This result indicates that the role of Asp54 in the oxidative half reaction is more prominent with regard to oxidase activity, consistent with the structure of wild-type PnFPOX, in which Asp54 assists in proper acceptance of oxygen at the position of the bound W2 in a plausible main oxygen channel.

Our structural comparison revealed that Asn56 in PnFPOX has a crucial role in assisting and stabilizing the oxygen path involving Asp54, Lys57, and Lys274 in terms of oxidase activity. This comparison also suggests that Asn56 in PnFPOX is essential for properly maintaining a plausible main oxygen channel and oxidase activity involved in the movement of Lys274. In addition, the results of the mutagenesis study provide direct evidence of decreased oxygen activity in PnFPOX N56A.

## Methods

### Protein sample preparation

The expression vector for His-tagged wild-type PnFPOX was constructed by PCR using the template plasmid pET28a_PnFPOX^8^, which contained primers designed with *Nde*I and *Xho*I recognition sequences (Forward: 5′-AAAACATATGGCGCCGTCCCGTGCA-3′; Reverse: 5′-TTTTCTCGAGCAGGTTCGCACGCGGCTTATCATGA-3′). The amplified products were cloned into the *Nde*I and *Xho*I sites of pET22b and the resultant expression vector pET22b_PnFPOX was used for expression of His-tagged wild-type PnFPOX. Mutant forms of PnFPOX were prepared by site-directed mutagenesis using the quick-change method with the primers listed in Supplementary Table S3 and pET22b_PnFPOX as the template.

Wild-type PnFPOX and the mutant N56A, D54A/N56A, and D54N/N56A were expressed in *E. coli* BL21 (DE3). The cells carrying the expression plasmid pET22b_PnFPOX, pET22b_PnFPOX N56A, pET22b_PnFPOX D54A/N56A, or pET22b_PnFPOX D54N/N56A were grown in Luria-Bertani (LB) medium containing 100 μg/mL ampicillin for 12 hours at 310 K as pre-culture, followed by growth in the medium containing 100 μg/mL ampicillin, 0.5% glycerol, 0.05% glucose, 0.2% α-lactose, 50 mM KH_2_PO_4_, 25 mM (NH_4_)_2_SO_4_, 50 mM Na_2_HPO_4_, and 1 mM MgSO_4_ for 24 hours at 298 K, after the addition of a 1% volume of the pre-culture. The cell lysate was prepared by centrifugation after disrupting the cells using a French press and purified by nickel affinity chromatography using a HisTrap column. The purity of the eluted protein was confirmed by Coomassie-stained SDS-PAGE, which showed a single band of 49 kDa and the concentration was measured using the DC Protein Assay Kit (Bio-Rad, Hercules, CA, USA). The purified protein solution was dialyzed against potassium phosphate buffer pH 7.0, overnight. The purified protein solution was concentrated to 7–13 mg/mL using an Amicon Ultra-X 30 kDa Ultracel (Merck Millipore, Billerica, MA, USA) for crystallization.

### Enzyme assay

The enzyme activities were measured as previously reported^18^.

In brief, FPOX activity was measured for oxidase and dehydrogenase activity. The kinetic parameters for wild type, N56A, D54N/N56A, and D54A/N56A were investigated using purified sample of each enzyme at 298 K in 10 mM potassium phosphate buffer (pH 7.0). The kinetic parameters for oxidase activities, (the formation of H_2_O_2_) were measured using1.5 mM 4-Aminoantipyrine (4-AA), 1.5 mM *N*-ethyl-*N*-(2-hydroxy-3-sulfopropyl)-3-methylaniline sodium salt (TOOS), and 2 U horseradish peroxidase ml^−1^. The formation of quinoneimine dye was measured spectrophotometrically at 555 nm. The kinetic parameters for dye mediated dehydrogenase activities (discolouring reaction occurred via the reduction of 2,6-dichlorophenolindophenol (DCIP)) were measured using 0.6 mM methylphenazinium methylsulfate (PMS) and 0.06 mM DCIP and was monitored at 600 nm. Substrate concentrations used for the investigations of oxidase activities were 0.1, 0.3, 0.5, 0.7, 1.0, 2.0 and 5.0 mM of f-^α^Val for wild type, 0.1, 0.3, 0.5, 0.7, and 1.0 mM of f-^α^Val for N56A, and 0.01, 0.03, 0.05, 0.07 and 0.1.mM of f-^α^Val for D54A/N56A and D54N/N56A. Substrate concentrations used for investigations of dye-mediated dehydrogenase activities were 0.1, 0.3, 0.5, 0.7, 1.0, 2.0 and 5.0 mM of f-^α^Val for wild type, N56A, D54A/N56A and D54N/N56A. The formation of quinoneimine dye and reduction of DCIP were measured at 546 and 600 nm, respectively. One unit is defined as the enzyme quantity that oxidizes 1 μmol of f-^α^Val per minute under the above reaction conditions. The representative fitting to determine kinetic parameter based on the Michaelis-Menten and Lineweaver-Burk plots is shown in Supplementary Information Figure S9 (for the oxidase activity of PnFPOX WT).

To compare the enzymatic activities of PnFPOX mutants with those of PnFPOX wild-type, partially-purified samples were prepared. The cultivated mutant cells were disrupted using BugBuster (Merck Millipore, Billerica, MA, USA). Further centrifugation (10,000 × g, 20 min, 277 K) resulted in removal of cellular debris. The obtained cell lysates were then used for the assay. The absorbance variation of each mutant was determined based on 1.0 mM substrate concentration. The concentrations of all mutants were measured as described above and SDS-PAGE analysis was conducted to confirm expression level of each samples (Supplementary Figure S5). The relative activities using partially-purified samples are summarized in Supplementary Table S1.

### Crystallization

The initial crystal screening was performed using the sitting drop vapor diffusion method and the mosquito system (TTP LabTech, Hertfordshire, UK). The protein concentrations of wild-type PnFPOX and N56A were 9.8 and 7.1 mg/mL, respectively. After a few days, yellow crystals were observed under several conditions using PEG/Ion 1 and 2, Index Screen I and II (Hampton Research Corp., CA, USA). To obtain well-diffracting crystals, crystallization conditions were optimized. Each of the protein solutions (0.8 µL) (wild-type, 9.8 mg/mL; N56A, 7.1 mg/mL) in 10 mM potassium phosphate buffer (pH 7.0) was mixed with the same volume of reservoir solution (8% (v/v) Tacsimate pH 5.0, 18–22% (w/v) PEG3350) against 50 μL of the reservoir solution using the sitting-drop method in a 96-well plate (Corning Inc., NY, USA) at 293 K. To prepare the PnFPOX and chloride complex, the crystals obtained from the above condition were soaked in a reservoir solution containing 2.5 M NaCl (within 1 minute).

### X-ray crystallography

A crystal of wild-type or N56A PnFPOX was mounted on a cryoloop and directly flash-cooled in a nitrogen-gas stream at 100 K. X-ray diffraction data were collected using the ADSC Quantum 270 CCD detector system on the Photon Factory Advanced Ring (PF-AR) NE3A beam lines in the KEK (Tsukuba, Japan). For crystals prepared with NaCl, X-ray diffraction data were collected using a Rigaku R-AXIS VII imaging system on a Rigaku RA-Micro7HF rotating anode (CuKα) X-ray generator with ValiMax optics (40 kV, 30 mA). Diffraction data were processed using HKL2000^25^, CrystalClear system (Rigaku Corp. Tokyo Japan), the XDS program^26^, and the CCP4 program suite^27^. The initial phases of wild-type PnFPOX were obtained by molecular replacement using the MOLREP^28^ program using the structure of FPOX obtained from *Eupenicillium terrenum* (PDB code: 4RSL) as a probe model. The following model building for protein was performed with the Autobuild program in the Phenix system^29^. Further model building and refinements were performed using the programs Coot^30^ and Refmac5^31^, respectively. Water molecules were introduced if peaks greater than 3.0 σ in the (*F*_o_ − *F*_c_) electron density map were in the range of a hydrogen bond. Structure validation was performed using the program PROCHECK^32^. The structure of PnFPOX N56A was determined by isomorphous replacement using the structure of wild-type PnFPOX. Data collection and refinement statistics are listed in Table 2 and Table S2. Figures 2, 3, 4, S1, S2, S3, S4, S6, S7, and S8 were drawn using the PyMol program (Schrödinger, LLC, New York, USA).

**Table 2:** Data collection and refinement statistics.

	Wild-type PnFPOX	N56A
Data collection		
Beamline	PF-AR NE3A	PF-AR NE3A
Temperature (K)	100	100
Wavelength (Å)	1.0000	1.0000
Resolution range (Å)	50.00–1.83 (1.86–1.83)	50.00–1.98 (2.01–1.98)
No. of measured refs.	114,981	78,434
No. of unique refs.	33,853	24,157
Redundancy	3.4 (3.3)	3.2 (3.0)
Completeness (%)	99.9 (99.9)	91.9 (84.7)
Mean *I*_*o*_/σ(*I*_*o*_)	6.1 (2.9)	7.8 (2.0)
*R*_*merge*_ (%)	13.6 (46.1)	8.6 (49.6)
Space group	*P*2_1_	*P*2_1_
Unit cell parameters *a, b, c* (Å) *β* (°)	*a* = 44.01	*a* = 43.58
	*b* = 93.4	*b* = 93.33
	*c* = 47.88	*c* = 47.76
	*β* = 97.48	*β* = 98.41
Refinement		
Resolution range (Å)	34.54–1.83 (1.86–1.83)	39.14–1.98 (2.03–1.98)
No. of refs.	31,660 (2,120)	22,908 (1,504)
Completeness (%)	98.3 (89.9)	91.8 (83.8)
*R*_*factor*_ (%)	14.2 (19.4)	15.8 (27.0)
*R*_*free*_ (%)	22.6 (27.5)	19.5 (36.2)
RMSD bond lengths (Å)	0.005	0.004
RMSD bond angles (°)	1.0	0.8
Ramachandran plot		
Most favoured region (%)	95.5	96.3
Additional allowed region (%)	3.8	3.0
*B*-factor (Å^2^)		
Protein	20.6	32.3
Ligand (FAD)	14.8	27.2
Ligand (ACY)	40.7 (3 molecules)	55.2
Water	30.7	37.0
PDB code	5T1E	5T1F

Values in parentheses are of the high-resolution bin. *R*_merge_ = Σ_*h*_ Σ_*i*_ [|*I*_*i*_(*h*) − <*I*(*h*) >|/ Σ_*h*_ Σ_*i*_ *I*_*i*_(*h*)], in which *I*_*i*_ i﻿s﻿ the *i*th measurement and <*I*(*h*)> is the weighted mean of all measurements of *I*(*h*).

### Protein channel analysis

For analysis of O_2_ trajectories across the structure, protein channels of the structures of wild-type PnFPOX and N56A were searched using the software program CAVER 3.0 (http://www.caver.cz/)^23^ with the default condition. The position of W1 forming a hydrogen bond with N5 of the isoalloxazine ring of FAD was selected as the starting point to retrieve accessible channels from the protein surface.

## Electronic supplementary material

Supplementary information
