# Supplementary material for: X-ray structures of fructosyl peptide oxidases revealing residues responsible for gating oxygen access in the oxidative half reaction
Source: Sci Rep. 2017 Jun 5;7:2790. doi: 10.1038/s41598-017-02657-5 (PMC5459902; doi:10.1038/s41598-017-02657-5)
Supplement: Supplementary file 1 — Supplementary information [file 41598_2017_2657_MOESM1_ESM.pdf]

X-ray structures of fructosyl peptide oxidases revealing residues responsible for gating oxygen access in the oxidative half reaction

Tomohisa Shimasaki[1 §], Hiromi Yoshida[2 §], Shigehiro Kamitori[2] & Koji Sode[1]*

[1]Department of Biotechnology and Life Science, Graduate School of Engineering, Tokyo University of Agriculture and Technology, 2-24-16, Nakamachi, Koganei, Tokyo 184-8588, Japan
[2]Life Science Research Center and Faculty of Medicine, Kagawa University, Ikenobe, Miki-cho, Kita-gun, Kagawa, Japan

*Corresponding author
Correspondence to: sode@cc.tuat.ac.jp

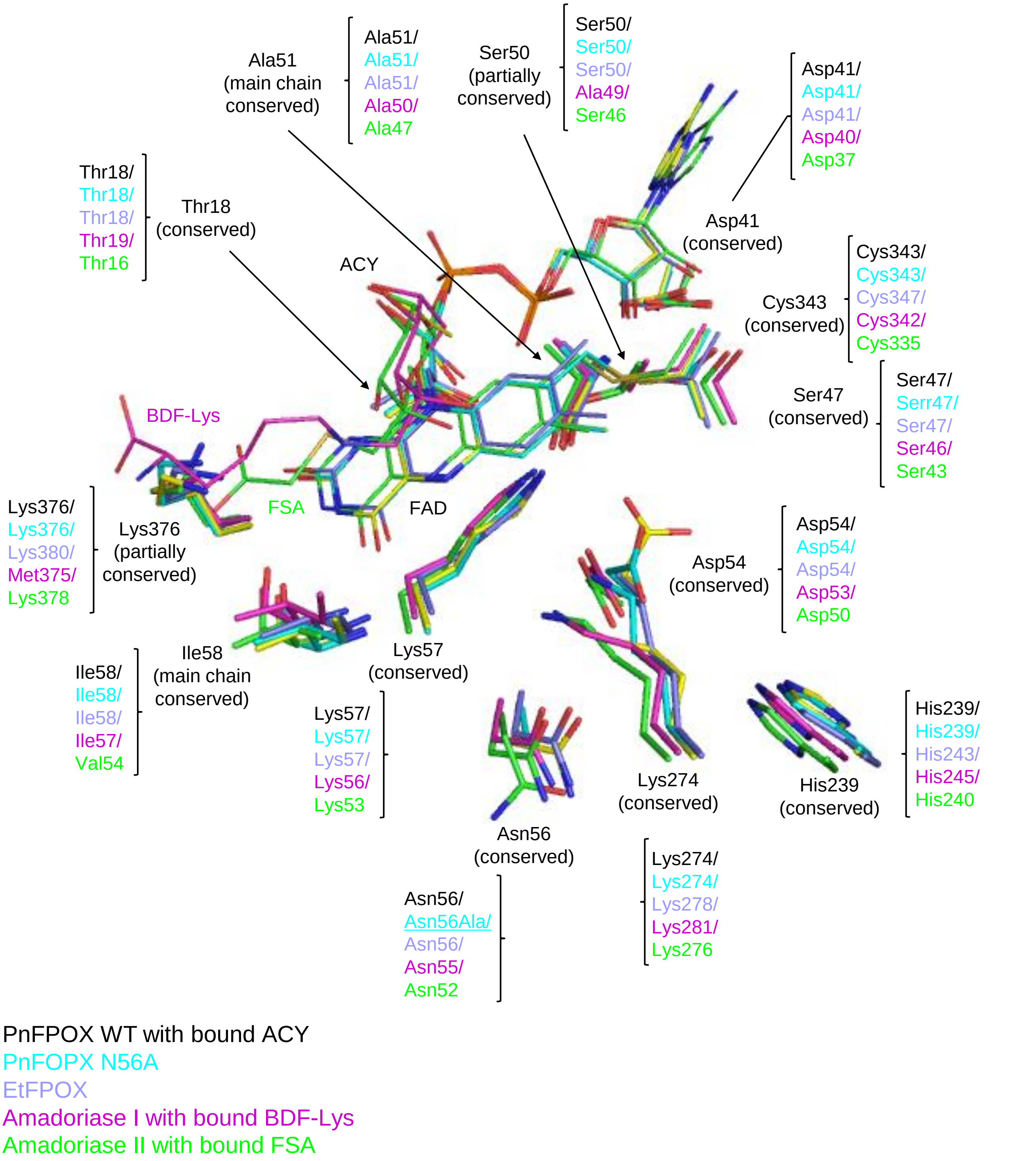

Supplementary Figure S1

Structural comparison around FAD at the *si*-face among PnFPOX WT with bound ACY (yellow), N56A (cyan), EtFPOX (light purple), amadoriase I with bound BDF-Lys (magenta) and amadoriase II with bound FSA (light green).

Six residues (Asp54, Asn56, Lys57, His239, Lys274, Cys343) in the vicinity of FAD at the *si*-face are structurally conserved in these enzymes. Especially, four of them (Asp54, Asn56, Lys57 and Lys274) are conserved in sequence alignment of eukaryotic FAOD/FPOX, according to Kim et al[12].

ACY: acetate
BDF: beta-D-fructopyranose
Lys: Lysine
FSA: 1-s-(carboxymethyl)-1-thio-beta-D-fructopyranose

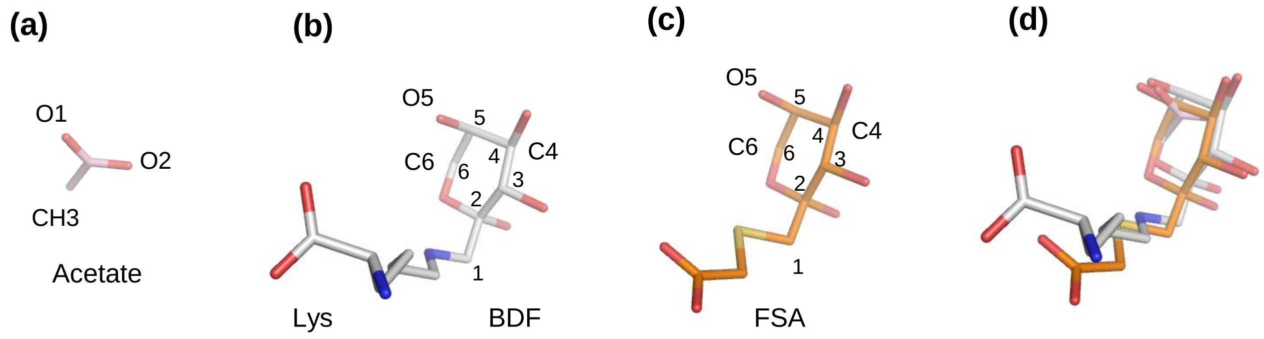

Supplementary Figure S2
The comparison of bound ligands in active site.
(a) The bound acetate in wild-type PnFPOX (b) The bound BDF-Lys in amadoliase I (PDB: 4XWZ) (c) The bound FSA in amadoriase II (PDB: 3DJE). (d) The bound ligands of (b) and (c) were superimposed onto (a).

BDF: beta-D-fructopyranose
Lys: Lysine
FSA: 1-s-(carboxymethyl)-1-thio-beta-D-fructopyranose

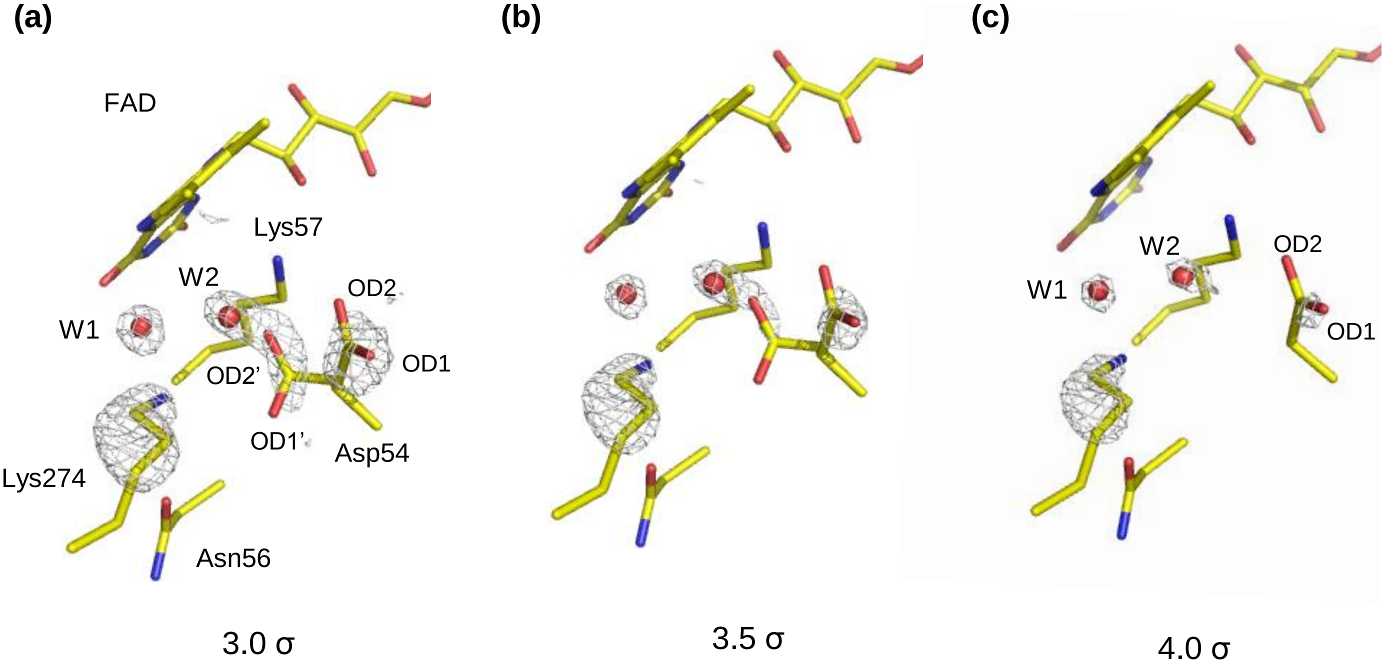

Supplementary Figure S3

SA-omit maps of the Asp54, Lys274, W1 and W2 in wild-type PnFPOX contoured at 3.0, 3.5, 4.0 σ (a, b, c, respectively) .

The possibility of the alternative conformation of Asp54 was shown, however, the distance between W2 and OD2’ of Asp54 in alternative conformation is 1.1 Å ((a), (b)). In the SA-omit maps at 4.0 σ, the positions of W2 and Asp54 were confirmed (c).

At the condition of 0.5 occupancy, the b-factors of OD1 and OD1’ (OD2 and OD2’) of Asp54 were 29.67 and 39.83 (27.99 and 37.33), respectively, and the b-factor of water molecule (W2) with 0.5 occupancy was 16.27. Although we chose the current position of Asp54 as main position in our current model from the SA-omit maps, the water molecule (W2) and the side chain of Asp54 could occupy the position alternatively.

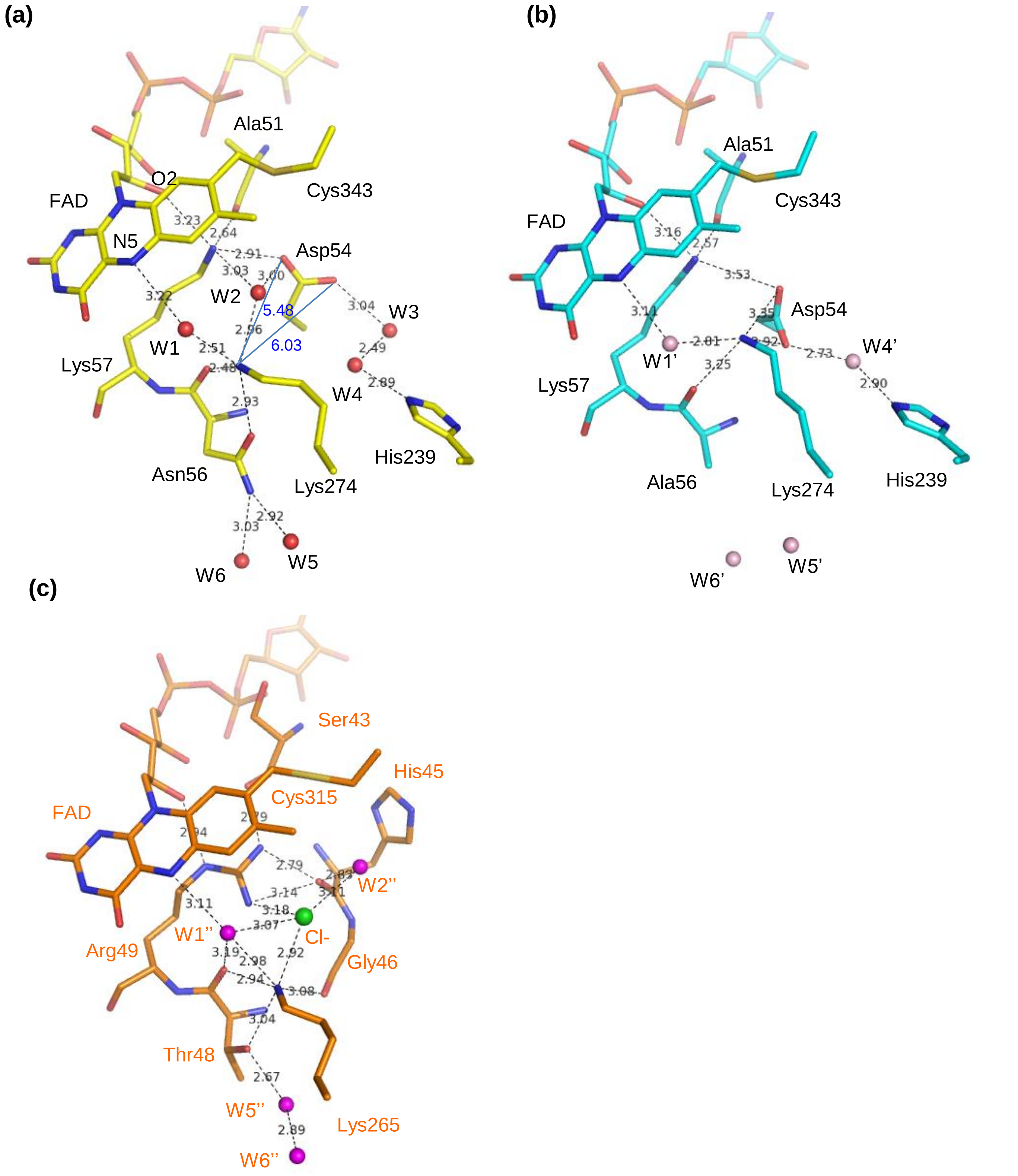

Supplementary Figure S4

Oxygen binding-site comparison between wild-type PnFPOX, N56A, and monomeric sarcosine oxidase (MSOX) with bound chloride.

(a) The structure of wild-type PnFPOX. Red spheres indicate water molecules. (b) The structure of PnFPOX N56A. Light pink spheres indicate water molecules. (c) The structure of MSOX with bound chloride (PDB: 3QSM). Light green sphere indicates chloride. Magenta spheres indicate water molecules.

Selected hydrogen bonds and salt bridge interactions (< 3.53 Å) are indicated by dotted lines with distances.

Blue solid lines indicate the distance between selected atoms.

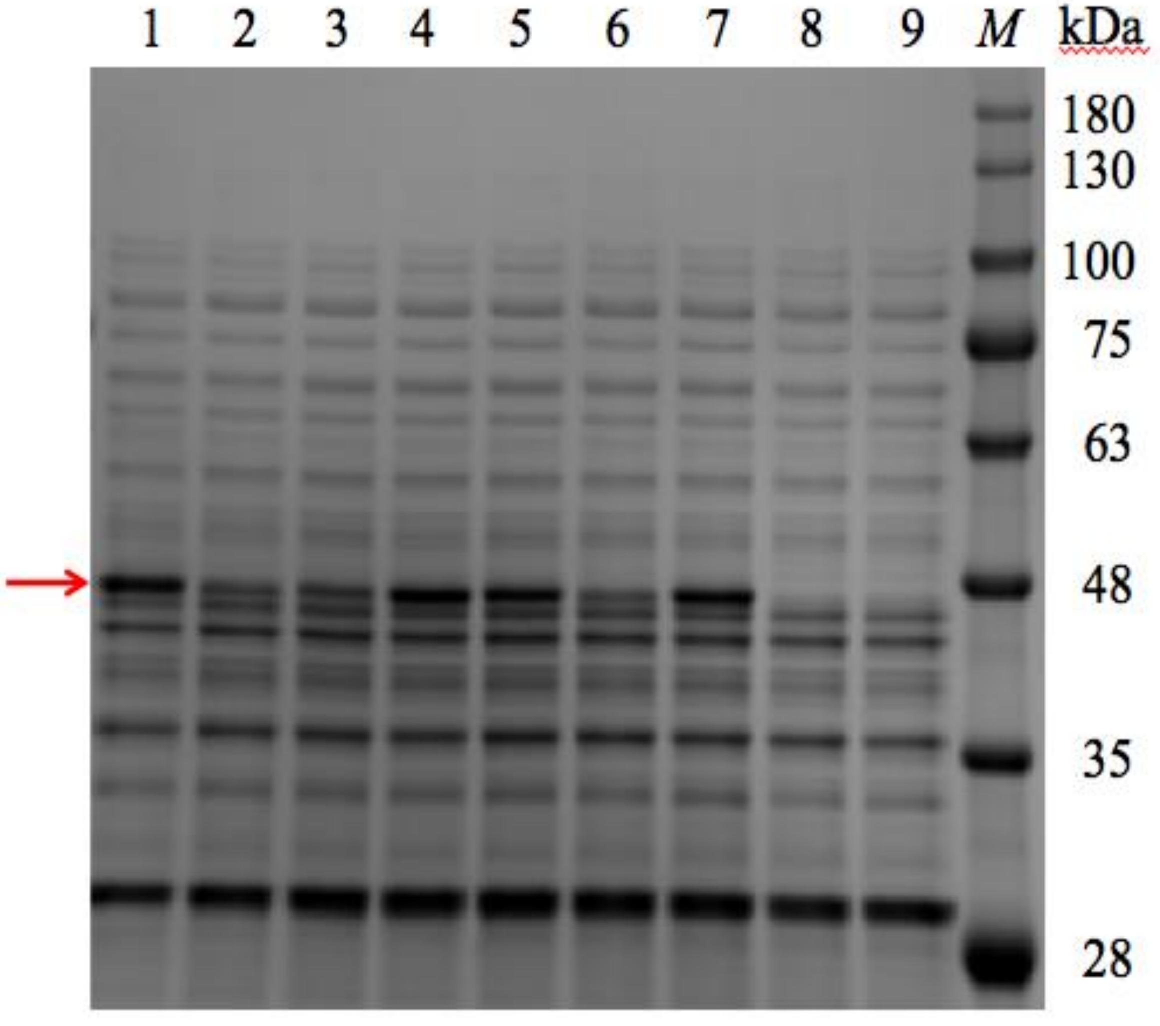

Supplementary Figure S5

SDS-PAGE analysis of water soluble fraction of cell lysate derived from recombinant *E.coli* expressing either wild-type or mutant enzyme.

Lane *M* contains molecular-mass markers. Water soluble fraction derived from cell lysate of *E.coli* expressing either wild-type or mutant enzyme, containing 7.5 μg was applied in each lane. The red arrow indicates the expected molecular weight (48kDa) of PnFPOX WT and mutants. Lane 1, WT; Lane 2, D54E; Lane 3, D54N; Lane 4, D54A; Lane 5 D54H; Lane 6, D54V; Lane 7, D54S; Lane 8, D54Y; Lane 9, D54F.

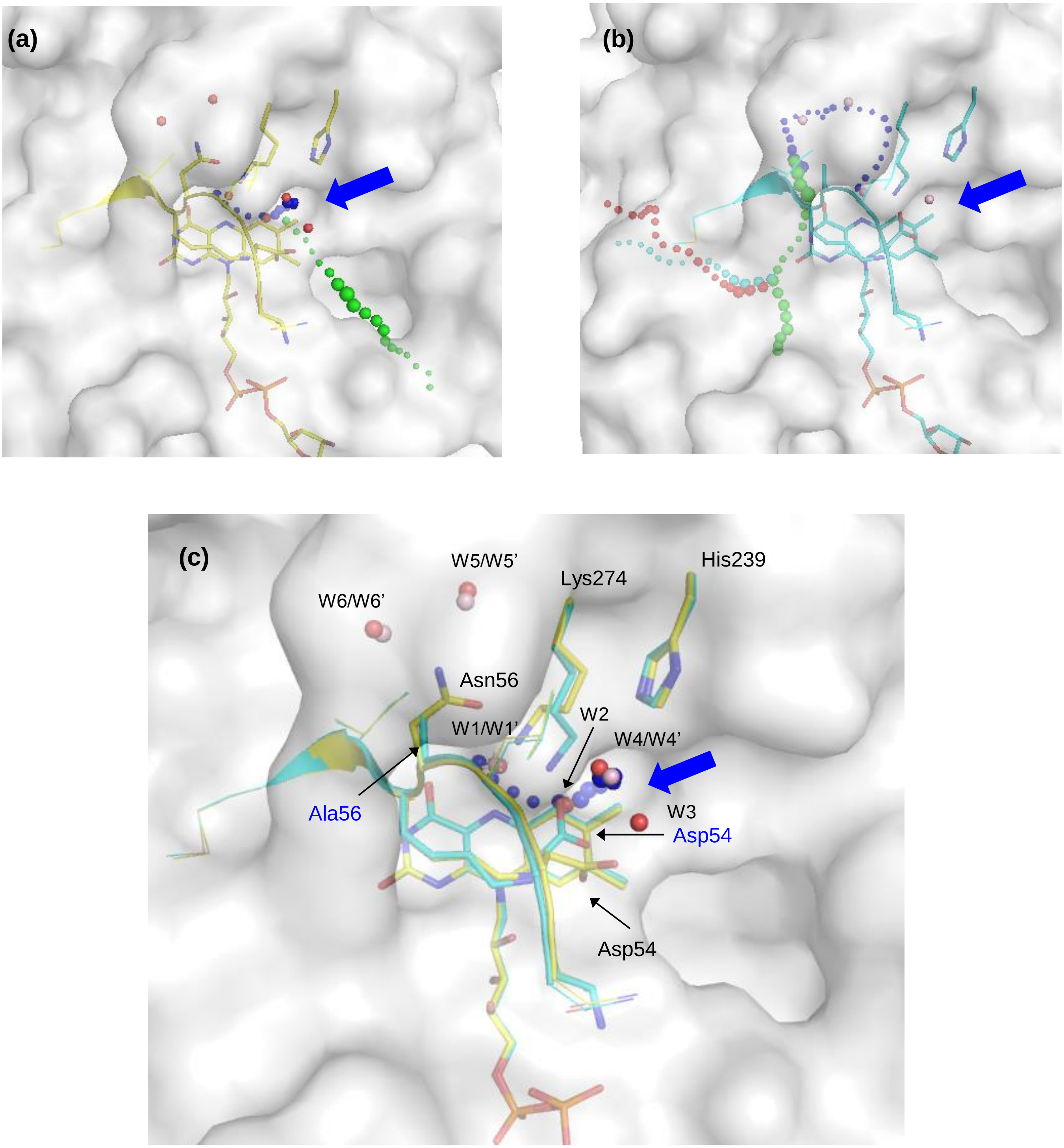

Supplementary Figure S6

Analysis of protein channels of wild-type PnFPOX and N56A

(a) Surface model of wild-type PnFPOX with oxygen accessible channels (light green and blue dotted spheres).

The entrance of short and plausible main channel is indicated with blue arrow.

(b) Surface model of PnFPOX N56A with oxygen accessible channel (blue, cyan, light green, and red dotted spheres). The entrance of short and plausible main channel in wild-type PnFPOX is indicated with blue arrow. The main channel is closed in PnFPOX N56A.

(c) Superimposition of the PnFPOX N56A (cyan stick) with water molecules (pink spheres) onto the surface model of wild-type PnFPOX (yellow stick) with water molecules (red spheres) and main oxygen accessible channel (blue dotted spheres with arrow).

In PnFPOX N56A, the movement of Asp54 and Lys274 blocks a short and plausible main channel observed in wild-type PnFPOX (blue dotted path with arrow). Another long path would enable oxygen to reach the active site instead of the closed path in PnFPOX N56A, but it would not be effective for competition with dye mediator for dehydrogenase activity.

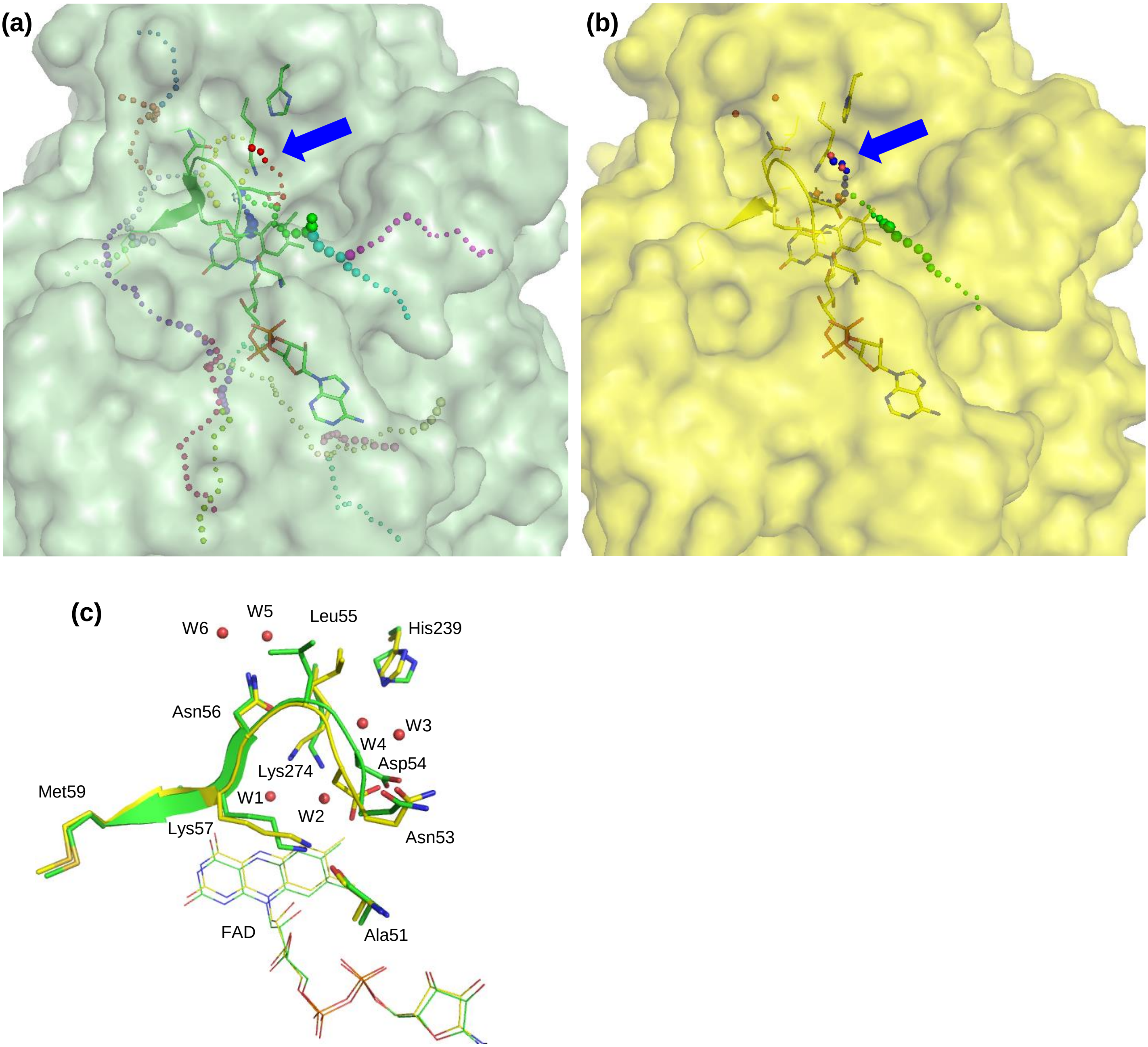

Supplementary Figure S7

Analysis of protein channels of wild-type PnFPOX in the presence/absence of NaCl

The surface models with oxygen accessible channels between the structures of wild-type PnFPOX in the presence (green stick and surface) / absence (yellow stick and surface) of NaCl were compared.

For comparison, the structure of Mol A in wild-type PnFPOX with NaCl was used, since the electron densities of the side chains of Asp54 and Lys57 were invisible in Mol B of wild-type PnFPOX with NaCl. The orientation of the side of Asp54 was not clear due to the weak electron density in Mol A. The entrance of short and plausible main channel is indicated with blue arrow.

(a) The surface model of wild-type PnFPOX with NaCl (green stick and surface). Oxygen accessible channels were shown in blue, light green, cyan, magenta, red, yellow, orange, purple, and other dotted spheres.

(b) The surface model of wild-type PnFPOX (yellow stick and surface) with water molecules (red spheres). Oxygen accessible channels were shown in light green and blue dotted spheres.

(c) Superimposition of the region (Asn53 – Met59) of the wild-type PnFPOX in the presence of NaCl (green stick) onto the corresponding region of the structure of wild-type PnFPOX in the absence of NaCl (yellow stick) and with water molecules (red spheres). Both FAD molecules were shown in line models.

In the vicinity of isoalloxazine ring of FAD in wild-type PnFPOX in the presence of NaCl (green stick), the region (Asn53 – Met59) including Asp54 and Lys57 was slightly moved, and the orientations of side chains of Asn53, Asp54, Leu55 were changed. Although the orientation of Asp54 was not clear due to the weak electron density, the orientation of Lys274 was also moved. The addition of NaCl caused a structural change of the region and gave more possible oxygen accessible channels leading to the putative main gate consisted a pair of Asp54 and Lys274.

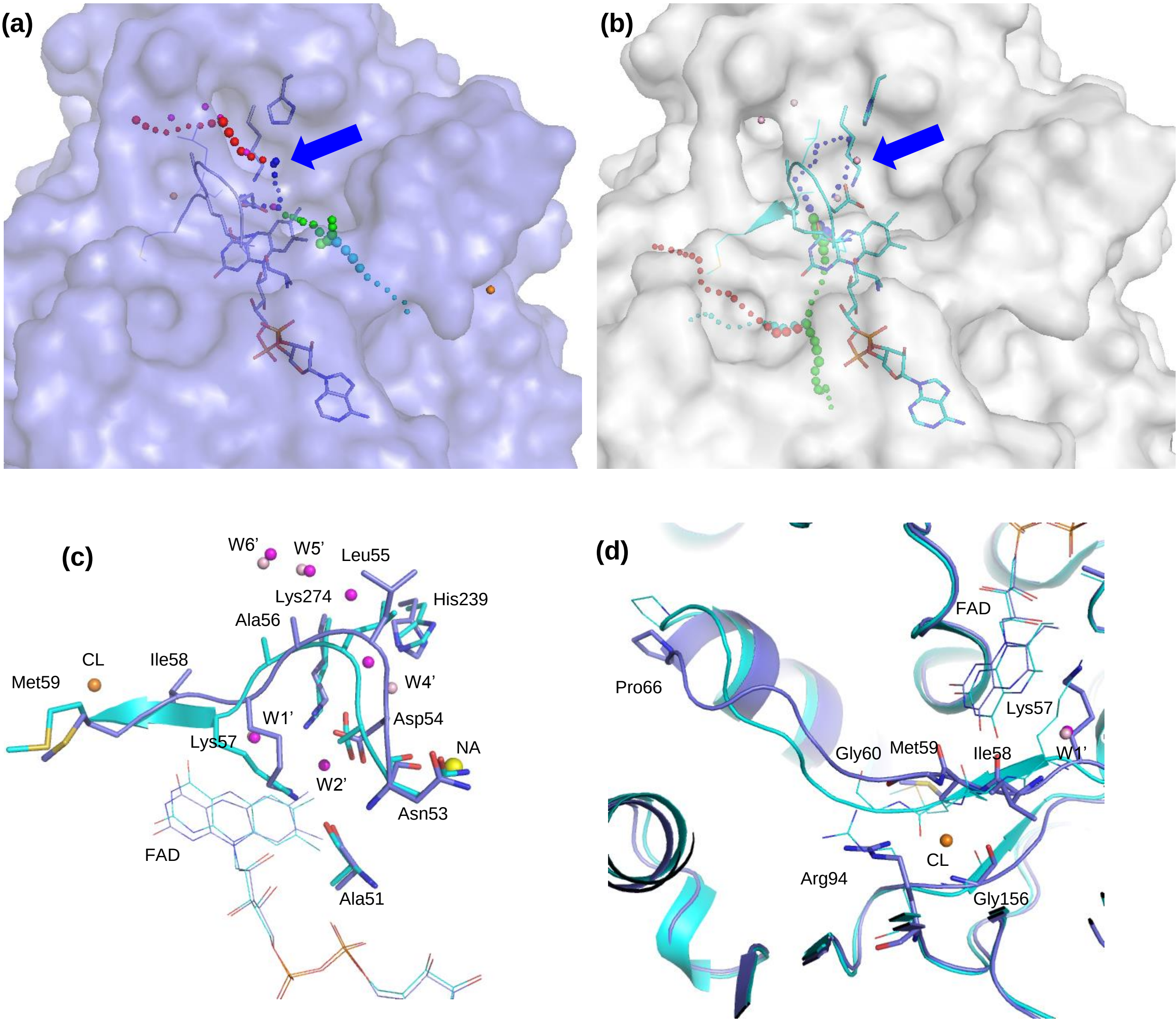

Supplementary Figure S8

Analysis of protein channels of PnFPOX N56A in the presence/absence of NaCl

The surface models with oxygen accessible channel between the structures of PnFPOX N56A in the presence (blue stick and surface) / absence (cyan stick and white surface) of NaCl were compared.

The region in the vicinity of FAD occurred conformational change in Mol A of PnFPOX N56A with NaCl, and the structure of Mol A was used for comparison to see any difference. The entrance of short and plausible main channel belonging to wild-type PnFPOX is indicated with blue arrow.

(a) The surface model of PnFPOX N56A in the presence of NaCl (blue stick and surface). Water molecules (magenta spheres) and oxygen accessible channels (blue, light green, cyan and red dotted spheres) were shown. The bound chlorides were shown in orange spheres.

(b) The surface model of PnFPOX N56A (cyan stick and white surface) with water molecules (pink spheres). Oxygen accessible channels were shown in blue, cyan, light green, and red dotted spheres.

(c) The region (Asn53 – Met59) of PnFPOX N56A in the presence of NaCl (blue stick) with water molecules (magenta spheres), the bound chloride (orange sphere) and the bound sodium (yellow sphere) was superimposed onto the corresponding region of the structure of PnFPOX N56A in the absence of NaCl (cyan stick) and with water molecules (pink spheres with the number). Both FAD molecules were shown in line models.

(d) Comparison of the structure in the vicinity of the bound chloride between PnFPOX N56A in the presence /absence of NaCl.

In the vicinity of the isoalloxazine ring of FAD, the region (Asn53 – Pro66) of PnFPOX N56A (cyan line) caused conformational change by addition of NaCl (PnFPOX N56A with NaCl, blue stick), giving the disorder of the side chains and shift of main chains. The bound chloride (orange sphere) was found in slightly opened area surrounded by Ile58, Met59, Gly60, Arg94, Gly156. The bound sodium interacted with carbonyl oxygen of Asn53. The addition of NaCl would create other plausible oxygen channels, but it caused the conformational change of the flexible loop region which might affect oxygen paths for oxygen activity.

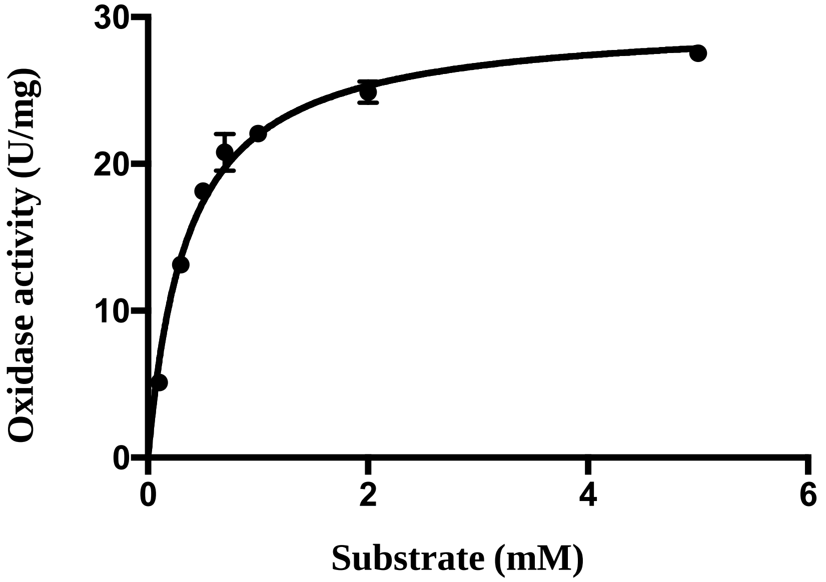

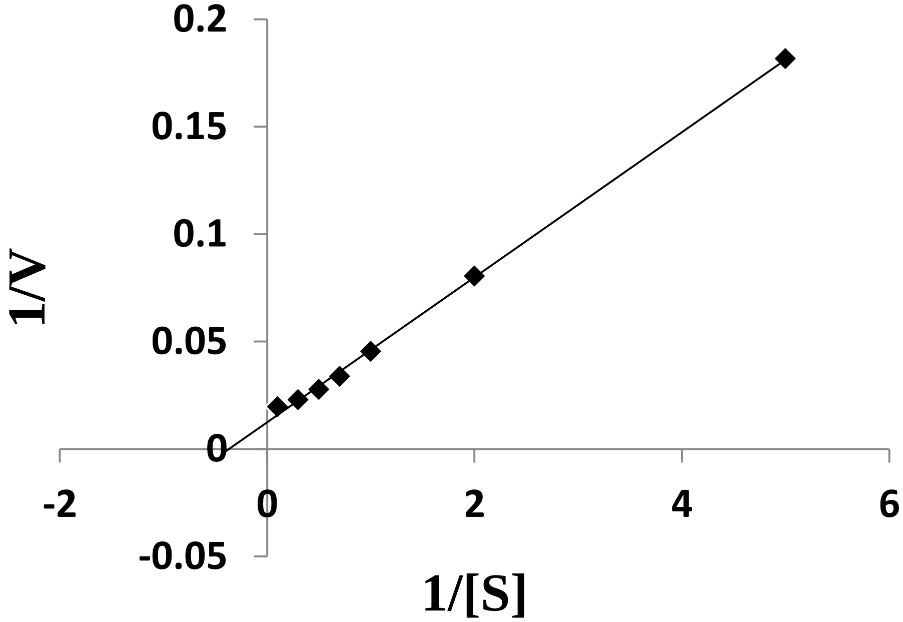

Supplementary Figure S9. Michaelis-Menten and Lineweaver-Burk plots of oxidase activity of PnFPOX WT.

Supplementary Table S1. Relative activities and Dh/Ox values of PnFPOX mutants

	Activity relative to wild-type PnFPOX (%)[1)]		Dh/Ox[2)]
	Oxidase activity	Dehydrogenase activity	
WT	100	100	0.59
D54E	1.2	7.5	3.7
D54N	1.8	6.0	1.9
D54A	5.1	14	2.1
D54H	3.0	8.8	2.3
D54V	0.11	6.6	46
D54S	4.1	9.5	1.8
D54Y	n.d.[3)]	0.64	n.a.[4)]
D54F	n.d.[3)]	0.97	n.a.[4)]

1) These values were calculated based on the oxidase or dehydrogenase activity of wild-type enzyme sample, which were prepared from cell lysate of recombinant *E.coli*. The values derived from wild-type enzyme were used as 100%.
2) Dh/Ox values were calculated based on the comparison of oxidase activities and dye-mediated dehydrogenase activities observed in the cell lysate of recombinant *E.coli* expressing either wild-type or mutant enzyme with 1.0 mM substrate concentration.
3) n.d. : not detected
4) n.a. ; not available

Supplementary Table S2. Data collection and refinement statistics.

	Wild-type PnFPOX with NaCl	N56A with NaCl
Data collection		
Beamline	MicroMax-007 HF	MicroMax-007 HF
Temperature (K)	100	100
Wavelength (Å)	1.5418	1.5418
Resolution range (Å)	19.80 – 3.08 (3.16 – 3.08)	19.64 – 1.80 (1.86 – 1.80)
No. of measured refs.	58,983	288,813
No. of unique refs.	16,927	80,576
Redundancy	3.5 (3.5)	3.6 (3.5)
Completeness (%)	96.7 (97.8)	96.0 (93.3)
Mean $I_o/\sigma(I_o)$	7.1 (4.0)	6.6 (1.8)
R_{merge} (%)	16.9 (47.6)	8.0 (51.4)
Space group	$P2_1$	$P2_1$
Unit cell parameters a, b, c (Å) β (°)	a = 45.52 b = 110.18 c = 96.34 β = 98.57	a = 45.12 b = 109.06 c = 95.02 β = 98.83
Refinement		
Resolution range (Å)	19.80 – 3.08 (3.16 – 3.08)	19.64 – 1.80 (1.85 – 1.80)
No. of refs.	16,073 (1,194)	76,559 (5,448)
Completeness (%)	96.8 (97.7)	96.0 (93.5)
R_{factor} (%)	25.1 (32.5)	20.92 (35.5)
R_{free} (%)	30.1 (38.6)	26.57 (40.6)
RMSD bond lengths (Å)	0.003	0.004
RMSD bond angles (º)	0.6	0.9
Ramachandran plot		
Most favoured region (%)	82.2	89.3
Additional allowed region (%)	16.3	9.8
B-factor (Å^2)		
Protein	29.5	32.5
Ligand (FAD)	18.2 (2 molecules)	24.2 (2 molecules)
Ligand (ACY)	-	55.7 (2 molecules)
Ligand (CL)	-	49.1 (5 molecules)
Ligand (NA)	-	40.8 (4 molecules)
Water	-	37.7
PDB code	-	5XAO

Values in parentheses are of the high-resolution bin. $R_{\text{merge}} = \Sigma_h \Sigma_i [|I_i(h) - \langle I(h)\rangle| / \Sigma_h \Sigma_i I_i(h)]$, in which I_i is the ith measurement and $\langle I(h)\rangle$ is the weighted mean of all measurements of $I(h)$.

Supplementary Table S3. Primer sequences for mutant construction

D54A sense	5'-CAATCTGCAGGCAATGCGCTGAATAAGATTATG-3'
D54A antisense	5'-CATAATCTTATTCAGCGCATTGCCTGCAGATTG-3'
D54E sense	5'-CAATCTGCAGGCAATGAACTGAATAAGATTATG-3'
D54E antisense	5'-CATAATCTTATTCAGTTCATTGCCTGCAGATTG-3'
D54F sense	5'-CAATCTGCAGGCAATTTCCTGAATAAGATTATG-3'
D54F antisense	5'-CATAATCTTATTCAGGAAATTGCCTGCAGATTG-3'
D54H sense	5'-AGGCAATCACCTGAATAAGATTATGGGCGTT-3'
D54H antisense	5'-AACGCCCATAATCTTATTCAGGTGATTGCCT-3'
D54N sense	5'-CAATCTGCAGGCAATAACCTGAATAAGATTATG-3'
D54N antisense	5'-CATAATCTTATTCAGGTTATTGCCTGCAGATTG-3'
D54S sense	5'-GCAGGCAATTCTCTGAATAAGATTATGGGCGTT-3'
D54S antisense	5'-AACGCCCATAATCTTATTCAGAGAATTGCCTGC-3'
D54V sense	5'-CAATCTGCAGGCAATGTTCTGAATAAGATTATG-3'
D54V antisense	5'-CATAATCTTATTCAGAACATTGCCTGCAGATTG-3'
D54Y sense	5'-CAATCTGCAGGCAATTACCTGAATAAGATTATG-3'
D54Y antisense	5'-CATAATCTTATTCAGGTAATTGCCTGCAGATTG-3'
N56A sense	5'-AGGCAATGACCTGGCGAAGATTATGGGCG-3'
N56A antisense	5'-CGCCCATAATCTTCGCCAGGTCATTGCCT-3'
